# What about vaginal extraction of the kidney? results of an online survey

**DOI:** 10.1590/S1677-5538.IBJU.2015.0170

**Published:** 2016

**Authors:** João Ferreira Cabral, Isaac Campos Braga, Frederico Branco, Vitor Cavadas, Avelino Fraga Ferreira, Miguel Silva Ramos

**Affiliations:** 1 Departamento de Urologia, Hospital de Santo António - C.H.P, Porto, Portugal;; 2 Instituto de Investigação em Ciências da Vida e da Saúde - I.C.V.S, Universidade do Minho; ICVS/3B´s - PT Laboratório Associado ao Governo, Braga, Portugal;; 3 Instituto de Ciências Biomédicas Abel Salazar - I.C.B.A.S - Universidade do Porto, Porto, Portugal

**Keywords:** Endoscopy, Kidney, Vagina

## Abstract

**Purpose:**

We aimed to characterize surgeons opinion about the vaginal extraction of the kidney after transperitoneal laparoscopic nephrectomy.

**Matherial and Methods:**

A 9-item questionnaire was published online (Survey Monkey ^TM^) and publicized via email to a multidisciplinary pool of surgeons in Portugal. Data was collected and statistical analysis was performed using IBM SPSS Statistics, Version 21.0.

**Results:**

Three hundred and fifty nine inquiries were sent, 154 surgeons completed the questionnaires (response rate of 43.0%). Fifty five point eight percent of the participants would choose the transvaginal approach for themselves or for a close relative. The most stated arguments were a better cosmesis (29.0%) expectancy of lower post operative pain (26.0%) and lower rate of incisional hernias (23.0%). Defenders of the transabdominal procedure justified with an expectancy of lower complication rate (39%), namely impairment of sexual function and fertility (22%). The female gender and the familiarity with transvaginal surgery were the stronger predictors of the option for this approach (70.6% vs 48.5%; p=0,016 and 85.3% vs 46.6%; p <0.001 respectively).

**Conclusions:**

Contrasting with similar surveys published on transvaginal NOTES, the vaginal specimen extraction after conventional laparoscopic nephrectomy was fairly accepted by the inquired surgeons.

## INTRODUCTION

Natural orifice transluminal endoscopic surgery (NOTES) is to be implemented for almost a decade. However, because of the lack of appropriate armamentarium and unproven safety, the technique presented in 2006 as “the new paradigm of surgery” (1), has hardly surpassed the initial barriers.

Contrarily, the natural orifices specimen **extraction** (NOSE) has proven to be feasible and safe (2-6) allowing the retrieval of surgical specimens after standard or mini laparoscopy.

Morcellation is another alternative for specimen retrieval without the need of wound enlargement; however, risks of intra-abdominal lesions, tumor seeding and impaired pathologic examination still elicit concerns in surgical community (7).

In the urological field, the first NOSE procedure was reported by Breda in 1993 (8), who first performed a transvaginal retrieval of a kidney specimen. In 2002 Gill reported a series of 10 laparoscopic radical nephrectomies followed by vaginal extraction (9) and in 2011 Alcaraz tested the safety of the procedure to the limit, reporting a series of 20 living donor laparoscopic nephrectomies with vaginal delivery (6).

However, the technique did not have a great spread, remaining confined to some high specialized centers (10).

We decided to conduct a survey directed to surgeons to better understand their opinion about the vaginal extraction of nephrectomy specimens.

## MATERIALS AND METHODS

We designed a survey in Portuguese language, consisting of a 9-item questionnaire (appendix) to evaluate five main items:

Personal and professional data, practice of laparoscopy, practice of transvaginal surgery, personal choice for kidney retrieval and justification of the option.

The inquiry was published online on a proper website (Survey Monkey ^TM^, Palo Alto, USA) and publicized via email to a multidisciplinary pool of surgeons, encompassing general surgeons, urologists and gynecologists, from three major surgical societies in Portugal.

No email reminders were sent in order to prevent re-answering.

Data was collected and statistical analysis was performed using IBM SPSS Statistics, Version 21.0 (IBM, New York, USA). Continuous data are expressed as mean and standard deviation. Chi square test was used for comparison of categorical variables with a significance level of 0.05.

## RESULTS

Three hundred and fifty nine inquiries were sent and 154 surgeons completed the questionnaires (response rate of 43.0%) (Table-1).

**Table 1:** Demographic and Professional Data.

Variable	N=154
**Age** (Mean ±SD)	39.52 (±10.7)
**Gender**	
Male	66.8%
**Differentiation**	
Specialist	59,7%
**Specialty**	
Urologists	44.2%
General Surgeons	31.2%
Gynecologists	24.7%
**Practice of Laparoscopy**	
No	16.2%
<20 annual procedures	35.7%
≥20 annual procedures	48.1%
**Practice of Transvaginal Surgery**	22.1%

Fifty five point eight percent of the respondents would choose the transvaginal approach for kidney retrieval for themselves or for a close relative.

**Figure 1:**
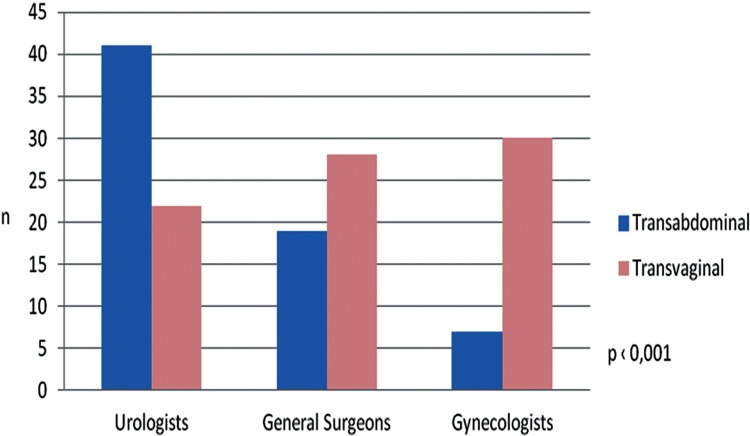
Surgeons option considering the area of specialization.

**Figure 2:**
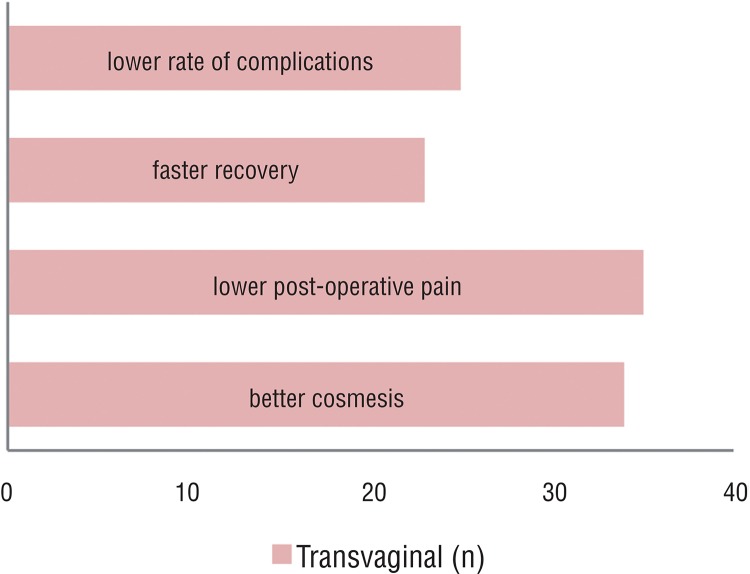
Mentioned arguments favoring the transabdominal approach.

The most stated arguments were a better cosmesis (29.0%) and expectancy of lower post operative pain (26.0%). Defenders of the transabdominal procedure justified with an expectancy of lower complication rate (39%), namely impairment of sexual function and fertility (22%) (Figures 1 and 2).

Female surgeons showed preference for the transvaginal access (70.6% vs 48.5%; p=0.016).

**Figure 3:**
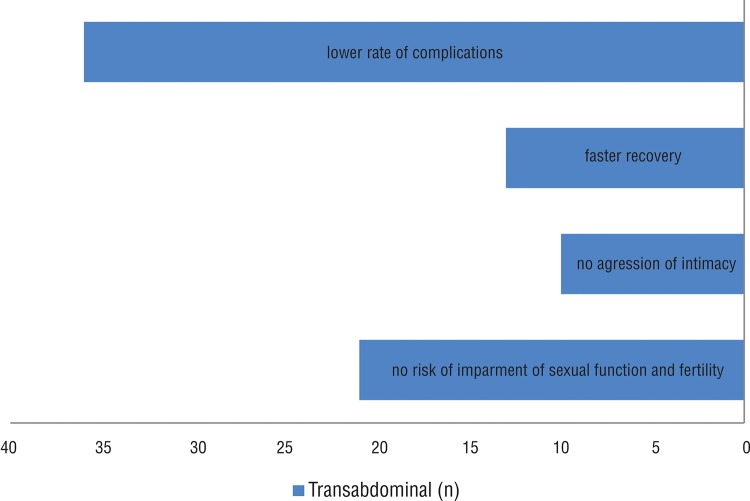
Mentioned arguments favoring the transabdominal approach.

Gynecologists and General surgeons were most likely to choose the transvaginal approach (81.5% and 60.4% respectively), while among the urologists only 38.2% would opt for this access (p<0.001) (Figure-3).

There was no difference in the option between residents and specialists (53.2% versus 57.6% respectively; p=0.71).

There was no statistical difference related with the reported number of laparoscopic procedures performed annually and the preference for the transvaginal approach.

The familiarity with transvaginal surgery was the strongest predictor of the option for this approach (85.3% vs. 46.6%; p<0.001).

## DISCUSSION

In our survey, the majority of the inquired surgeons privileged the transvaginal extraction of nephrectomy specimens over its transabdominal counterpart.

Gynecologists were the most enthusiasts, which is coherent with the fact that gynecologists perform transvaginal surgery for decades. In fact, 90% of the surgeons that stated practice of transvaginal surgery were gynecologists.

Urologists were the least like to choose the transvaginal approach. A possible explanation may be related with the number of patients amenable for vaginal extraction, which, in the majority of centers is probably too small to sensitize surgeons and to justify specific training. Excluding most of the tumors, because of specimen size, and living donors, which are only performed in specific centers, the majority of urologists can only count on excluded kidneys to get experience on vaginal extraction.

Overall, the expectation of lower post operative pain and better cosmesis were the most mentioned arguments by enthusiasts of the transvaginal retrieval, which is in general agreement with results of similar surveys on NOTES and LESS (11).

The defenders of the conventional abdominal extraction justified their option with the expectation of lower complication rate and expressed concerns about sexual function and fertility. These concerns are probably restraints to widespread acceptance of transvaginal surgery. Although, studies have shown that per-operative complications are negligible when the transvaginal access is created under direct vision (12) and infection is a rare event (≤1%) (12, 13). Sexual function seems not to be affected by the transvaginal access while fertility questions are more controversial. (14-17).

Tanaka M, et al. assessed the long-term complications, including infertility, after transvaginal peritoneal surgery in a group of young patients. They found no evidence that this approach caused infertility or dyspareunia (18).

Female surgeons preferred the transvaginal approach, which is in agreement with similar studies conducted on NOTES, (19-21) that showed that the majority of the inquired women would opt for the transluminal procedures, not only because of the better cosmesis but also expecting a lower post operative pain and lower risk of hernia formation. Probably women see naturally the vagina as a possible route of organ delivery.

Although surgeons with practice in laparoscopy tended to be more prone to choose the vaginal route, it was the experience in vaginal surgery that clearly prompted surgeons to opt for this approach. This suggests that surgeons experienced in vaginal surgery find the vaginal extraction safe and advantageous, and on the other hand, is natural that surgeons with no experience in vaginal surgery tend to “fear” this approach.

Our results also show contrasts with similar studies conducted on NOTES. In the article published by Thele, (22) whereas 69.2% of the inquired surgeons classified transvaginal NOTES as acceptable, only 32.7% considered the procedure appropriate for abdominal surgery and just 28.8% would recommend it. Concerns with infection, visceral lesions and infertility were the most expressed arguments. Volckmann and collaborators surveyed the members of three major surgical societies; 23% respondents demonstrated a great interest on NOTES, however, only 26% would personally undergo a NOTES procedure. Safety was considered the most important factor in the option. Probably, NOTES and NOSE, do not elicit the same concerns in the surgical community.

Public perception of new surgical procedures may not always consider all their potential risks and benefits; however it is well known the importance of public demanding in the evolution of surgical techniques. Population based surveys found a great acceptance of female public for the transvaginal procedures once safety is assured. In the study of Peterson et al. 73% of the respondents would consider a transvaginal procedure and 68% would opt for the procedure if safety was equivalent to laparoscopy (20). Olakengil et al. surveyed female living donors about transvaginal NOTES nephrectomy; 51% would have opted for this approach if safety was the same (23).

The present study is the first evaluating surgeons opinion on NOSE, however, it is has some limitations, namely, the use of a non validated questionnaire and the national character of the survey.

## CONCLUSIONS

The transvaginal kidney retrieval was the approach of choice of the majority of the inquired gynecologists and general surgeons but not to the urologists.

The lack of experience in vaginal surgery and the apprehension of long-term effects on sexual function and fertility can be obstacles to the widespread of this technique.
